# A Review of Developments in Carbon-Based Nanocomposite Electrodes for Noninvasive Electroencephalography

**DOI:** 10.3390/s25072274

**Published:** 2025-04-03

**Authors:** Hector Medina, Nathan Child

**Affiliations:** School of Engineering, Liberty University, University Blvd, Lynchburg, VA 24515, USA; nchild@liberty.edu

**Keywords:** wearables, biosensors, polymer nanocomposites, carbon nanoparticles, tunable properties, electroencephalography

## Abstract

Wearable biosensors have been of interest for their wide range of uses, varying from recording biological signals to measuring strain of bending joints. Carbon nanoparticles have been utilized in biocompatible polymers to create nanocomposites with highly tunable mechanical and electrical properties. These nanocomposites have been demonstrated to be highly effective as wearable sensors for recording physiological signals such as electroencephalography (EEG), offering advantages in mechanical and electrical properties and signal quality over commercially available sensors while maintaining feasibility and scalability in manufacturing. This review aims to provide a critical summary of the recent literature on the properties, design, fabrication, and performance of carbon-based nanocomposites for EEG electrodes. The goal of this review is to highlight the various design configurations and properties thereof, manufacturing methods, performance measurements, and related challenges associated with these promising noninvasive dry soft electrodes. While this technology offers many advantages over either other noninvasive or their invasive counterparts, there are still various challenges and opportunities for improvements and innovation. For example, the investigation of gradient composite structures, hybrid nanocomposite/composite materials, hierarchical contact surfaces, and the influence of loading and alignment of the dispersal phase in the performance of these electrodes could lead to novel and better designs. Finally, current practices for evaluating the performance of novel EEG electrodes are discussed and challenged, emphasizing the critical need for the development of standardized assessment protocols, which could provide reliability in the field, enable benchmarking, and hence promote innovation.

## 1. Introduction

**Figure 1:**
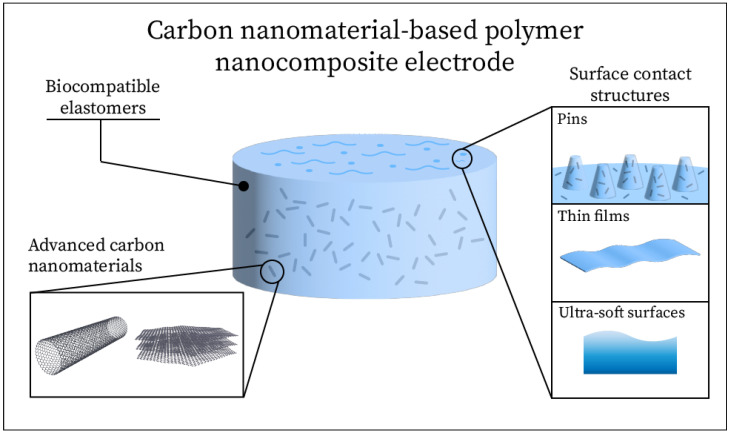
Visual abstract of a carbon nanomaterial-based nanocomposite electrode for noninvasive EEG.

In recent years, carbon nanomaterial-based nanocomposites have been of great interest for the development of wearable sensors [1,2,3,4]. In particular, carbon nanotubes (CNT), carbon nanofibers (CNF), and various forms of graphene have been utilized to create flexible sensors with advanced mechanical and electrical properties [1,5,6]. For a sensor to be effective as a long-term wearable monitoring device, it must be, at least, safe (e.g., biocompatible with skin), comfortable (e.g., soft and compliant to the skin surface) while maintaining sufficient and reliable transduction characteristics [7,8]. Soft, flexible sensors can be fabricated using elastic polymers (e.g., elastomers) as a substrate matrix, into which nanomaterials of various sizes and shapes may be embedded or arranged in various patterns, depending on the sensor’s intended function. This approach can yield biocompatible, comfortable sensors that conform to human skin for optimal signal detection and that have been limitedly employed in physiological signal monitoring [3,9,10,11,12,13]. In particular, these electrodes have been used for noninvasive electroencephalography (EEG) with some success, achieving low impedance, high conductivity, and reduced signal artifacts [14,15,16,17,18,19]. See Figure 1.

Current commercially available electrodes, which exist under the categories of invasive, noninvasive wet, and noninvasive dry, still face many challenges. Invasive electrodes require extensive surgical procedures to be implanted beneath the scalp, which can be expensive, inaccessible for many people, and can have undesirable complications [20,21,22]. Wet electrodes provide a good alternative to invasive electrodes, achieving high signal quality and low impedance using saline or gel to increase conductivity, but they have lengthy setup and clean-up processes and can be impractical outside of a clinical setting [23,24,25,26]. They can also be uncomfortable and unreliable for long-term usage due to a liquid or gel pressed against the user’s skin, which dries out over time, reducing signal quality [24,25,26]. Dry electrodes are also noninvasive and can achieve conductivity without a fluid using conductive coatings over a rigid surface, which makes them optimal for use outside of a clinical setting. Despite these advantages, they tend to have much higher impedance, are much more prone to signal artifacts from muscles or physical shifts across the surface, and can be uncomfortable or even painful to the wearer due to their rigid and pronged designs [24,25,26]. Therefore, the idea of a soft, comfortable, and dry EEG electrode that can achieve a high signal-to-noise ratio (SNR), high conductivity, and impedance equal to or lower than wet electrodes is of great appeal. In this review, literature that explores carbon-based nanocomposite EEG electrodes in recent years is explored and consolidated.

In contrast, other reported reviews have discussed instead carbon-based materials for biosensors [4,27], conductive polymer-based nanostructured electrodes [28], carbon nanotube-based wearable sensors [1,29] or other advanced carbon nanomaterials [2]. However, there are no existing reviews, to the best of our knowledge, that focus on carbon-based nanocomposite electrodes for EEG specifically. As this is a promising concept for EEG electrodes, this review aims to compile the existing literature and provide insight for future work in the field of carbon-based nanocomposite EEG electrodes. Some of the previously cited reviews do mention the application of these materials as electrophysiological sensors or EEG electrodes for brain-computer interface (BCI), but only as a subsection of a much larger review [27], which renders them lacking important information. Therefore, this work focuses on the intricacies and challenges related to the design and fabrication of sensors specifically designed for noninvasive EEG recording from the scalp using carbon-based nanocomposite materials. Comparison against some other technologies, when included, is for the purpose of contrasting and highlighting the differences.

This work is outlined as follows. Developments in the design and manufacturing methods of the electrodes are first discussed, comparing the various methods in which carbon nanomaterials are utilized in the dispersed phase of the nanocomposite and optimized specifically for EEG electrodes. Next, the mechanical and electrical properties of the EEG electrodes are discussed, as well as aspects related to biocompatibility and user comfort. Finally, we discussed opportunities for improvement and development of innovative technologies, as well as some opportunities for future research.

## 2. Electrode Design

When designing a noninvasive, carbon-based electrode for electrophysiological signal recording (such as EEG), several key factors must be taken into account. One of the first considerations is selecting the type of conductive carbon nanomaterial(s) to incorporate, as carbon nanomaterials come in various forms and offer superior electrical and mechanical properties [2,30,31]. Another important decision is whether to rely solely on a carbon nanomaterial for conductivity or to employ a hybrid approach by combining carbon with other conductive nanomaterials. Other materials, such as silver (Ag), gold (Au), and titanium dioxide (TiO_2_) nanomaterials, can be used in addition to carbons to fine-tune the electrode properties [32,33,34,35,36,37,38]. A substrate must then be selected that can integrate the carbon nanomaterials in order to function as an electrode. These substrates commonly include elastic polymers [15,39,40,41] or other flexible materials [42,43,44], and are typically selected to fit a specific application. Finally, a surface contact structure may be integrated into the electrode design to allow it to achieve conformal contact with the curves of the human skin surface. In particular, for a noninvasive EEG electrode to be effective on the scalp, it may achieve better skin contact with structures designed to reach through the hair down to the skin surface [18,38,45,46]. By taking each of these factors into consideration when designing the EEG electrode, it may be possible to design a new dry, noninvasive, and low-cost EEG electrode that will outperform the current commercial standards.

### 2.1. Carbon Nanomaterials

Carbon nanomaterials are known to exist in zero-, one-, and two-dimensional (0D, 1D, 2D) forms which can agglomerate to form three-dimensional (3D) carbon nanomaterials [30,31,47]. These nanomaterials have very desirable properties for electrode fabrication, including high surface area, electron transmission, electrical conductivity, and mechanical strength [31,48], causing them to be widely used for electrophysiological signal recording. When fabricating EEG electrodes, the most commonly utilized carbon nanomaterials are carbon nanotubes (CNT), carbon nanofibers (CNF), graphene sheets, and carbon black. While most recently published articles utilize nano-scale carbon materials, some publications use milli- and micro-scale materials such as carbon fibers and carbon particles to fabricate composites [18,38,46,49,50,51]. These articles have been included as they provide perspective on how larger carbon materials may affect the electrode properties. A summary of the general characteristics of the reviewed carbon nanomaterials is provided in Table 1.

#### 2.1.1. Carbon Nanotubes

**Figure 2:**
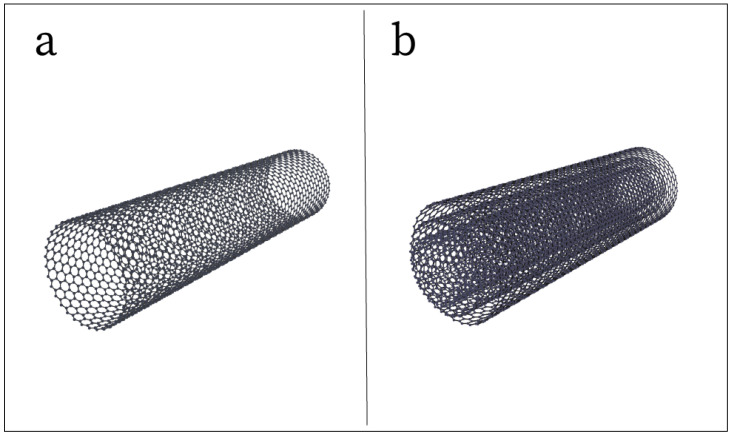
Illustrations of the structures of (**a**) single-walled carbon nanotubes and (**b**) multi-walled carbon nanotubes.

A carbon nanotube can be defined as one or more seamless cylindrical shells of graphitic sheets [58]. In other words, they are hollow, cylindrical tubes that are derived from sheets of graphene. There are two main types of CNTs: single-walled CNT (SWCNT) and multi-walled CNT (MWCNT). SWCNTs are comprised of a single graphene sheet wrapped to form a cylindrical tube (Figure 2a), while MWCNTs are comprised of an array of concentrically nested graphene sheets, forming a multi-walled tube (Figure 2b) [59]. This nanomaterial has been used primarily in two different methods for electrode fabrication, being CNTs as a conductive filler in an elastic polymer matrix [15,18,33,37,38,39,41,51,60,61,62,63,64,65], and using CNTs to coat a conductive layer on the surface of a substrate [66,67,68]. The more common method of integrating CNTs into the electrode design involves dispersing bulk CNTs into either a prepolymer or an uncured polymer mixture and using a manual or automatic mixing method to disperse the filler through the polymer and create a homogeneous mixture. The mixture can then be poured into molds and cured to create conductive polymers that can function as electrodes. To achieve consistent, superior electrical and mechanical properties, it is imperative that the filler is dispersed homogeneously throughout the polymer before the curing process takes place. This can be a challenge due to the initial state of CNTs, which is that of highly bundled and tangled aggregates due to inter-tube van der Waals attractive forces [69,70]. These bundles can be separated through physical approaches such as sonication, mechanical stirring, and milling that transfer physical shear stress to the nanotubes, breaking down the bundles [69]. Sonication is frequently selected as the method to break apart and homogeneously disperse the CNTs through a polymer mixture for electrode design, as it has repeatedly been demonstrated to be highly effective [15,33,37,60,61,62]. The dispersion process can be aided by adding a solvent, such as isopropyl alcohol (IPA) [15,37,61,62] or toluene [60], to the mixture which both dissolves the polymer and causes the nanotubes to disperse more easily [69]. Once the mixture is homogeneous, the solvent can be evaporated out, leaving only the CNTs dispersed through the polymer.

The homogeneously dispersed nanotubes create a dense network of conductive pathways throughout the polymer, enabling high conductivity and efficient electron transmission [39]. A mechanical stirring method can also be used to break down and disperse the CNT bundles, such as paste mixers [39], magnetic stirrers [38,60], and manual stirring by hand. Less frequently used dispersion methods for electrode fabrication include milling [39,65] and dispersion by shear flow [65]. The less common method of integrating CNTs into the electrode design involves using CNTs to create a conductive layer on the surface of the electrode substrate. This method may be selected when microstructures or other complex geometries are desired in the electrode design. One such design that utilizes a conductive CNT layer is that of aligned carbon nanotube (ACNT) sheets to further increase electron transmission efficiency [66]. The ACNT membranes are drawn from a CNT forest, and the membrane is then stacked on a nonadhesive substrate until a desired number of layers is reached. The stacked ACNT sheets can then be transferred to a flexible, adhesive substrate and effectively used as a self-adhesive EEG electrode. The alignment of nanotubes within the ACNT sheets allows for electron transfer at a much more efficient rate than that of randomly oriented CNTs [71]. Alternatively, the CNTs can be dispersed in a solvent with previously discussed methods and spin-coated [67] or spray-coated [68] to form a thin, flexible conductive layer as part of the electrode design. A summary of CNT integration in EEG electrode design and fabrication is presented in Table 2, including utilization, dispersion methods, and CNT shape and dimensions if the information was provided by the authors; ’CNT’ is used if single- versus multi-walled was not specified.

#### 2.1.2. Carbon Nanofibers

**Figure 3:**
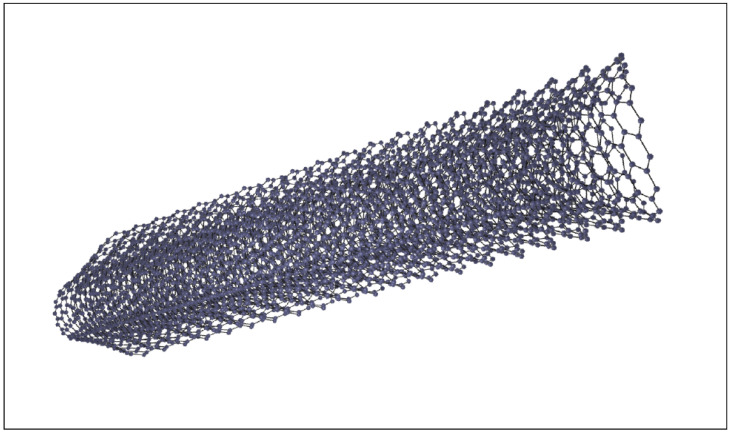
Illustration of a conical carbon nanofiber.

Carbon nanofibers (CNF) are similar to CNTs in that they are both 1D carbon nanomaterials with diameters that range from 1 nm to 100 nm and lengths that can range from 10 nm to a few centimeters [31,72]. The main difference between the two lies in their morphology, as CNFs are cylindrical nanostructures with graphene layers arranged as stacked cones, cups, or plates (Figure 3), while CNTs more closely represent hollow tubes. CNFs will generally tend to be larger than CNTs and are synthesized at lower temperatures [31,72]. Because of their similar properties, larger geometry, and less expensive synthesis, CNFs may be a more cost-effective alternative to CNTs for EEG electrode fabrication. CNFs combined with carbon black (CB) were demonstrated to be an effective conductive filler in a sorbitol-modified polyborosiloxane (SPBS) matrix to form a viscoelastic dry skin-like electrode that can adapt to hairy skin and achieve conformal skin contact [73]. The CNF and CB were mixed with a weight ratio of 7/3 and dispersed in chloroform via sonication. SPBS was added to the dispersion, and further sonication was applied to create a homogeneous mixture, which could then be molded into various electrode shapes. Furthermore, CNFs were demonstrated to be capable as the sole conductive filler material in a PDMS matrix to create a CNF-PDMS conductive elastomer electrode for EEG recording [17]. CNFs were mixed into the PDMS solution at ratios from 1 to 7 vol%, and manual mixing was preferred at volume fractions above 3% when the mixture became paste-like and could not be poured. When the filler ratio increased significantly beyond the material’s electrical percolation threshold, the electrode experienced a greatly reduced electrical dependence on compression; additionally, the electrode’s mechanical properties quickly decreased as the filler ratio increased, becoming stiffer and less conformal to the skin. While CNF has been utilized less than CNT for EEG electrode fabrication, it shows high potential as a lower-cost alternative to CNT for the fabrication of conductive nanocomposites. CNF can be utilized in very similar methods to CNT for EEG electrode fabrication, though they may result in less desirable mechanical and electrical properties due to their larger, less uniform morphology.

#### 2.1.3. Graphene

**Figure 4:**
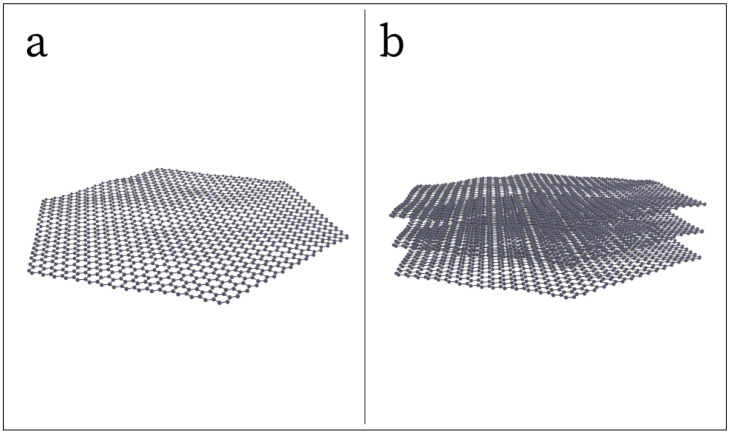
Illustrations of (**a**) monolayer graphene and (**b**) few-layer graphene.

Graphene is defined as a flat monolayer of carbon atoms arranged in a 2D densely packed hexagonal lattice, and it is the basic structure that other 0D, 1D, and 3D carbon nanomaterials are derived from [55,74]. For example, carbon nanotubes are considered a 1D nanomaterial and are formed by a 2D hexagonal lattice rolling into a tubular shape [55]. The material can exist as single-, double-, and few-layer (3 to <10) graphene [55], and it displays excellent mechanical, electrical, and thermal properties [31], offering a wide variety of potential applications. In particular, graphene has been utilized in its various monolayer and layered states in EEG electrode fabrication not only for its advanced electrical properties but also for its stability and high biocompatibility for on-skin applications [5,75]. Due to its very thin nature, graphene is commonly utilized to create highly conductive and very thin electrodes that conform to the skin surface [13,35,36,40,76,77,78,79,80]. One-atom-thick monolayer graphene (Figure 4a) has been utilized as a conductive layer, being deposited onto the surface of thin (<100 μm) substrates to achieve ultra-high skin-surface conformation, which not only enhances the signal quality of the resultant electrode but also makes the electrode much more comfortable for the user [13,79]. Bilayer graphene electrodes have also been fabricated by encasing a second material, such as the conductive polymer poly(3,4-ethylenedioxythiophene):poly(styrenesulfonate) (PEDOT:PSS) [76] and molybdenum chloride (MoCl_5_) [36]. Few-layer graphene (Figure 4b) and 3D graphene structures have also been used to form thin, flexible films that can conform to the skin surface, but they tend to be less optically transparent than mono- and bilayer graphene electrodes [35,40,77,78,80]. Aside from thin, skin-like electrodes, graphene can also be used to form conductive layers as one of the components of an electrode’s overall design. The methods to deposit a graphene layer to the electrode surface include chemical vapor deposition (CVD) [16,32], physical vapor deposition (PVD) [81], dip coating [44], catalytic alloy growth [82], and solidifying a layer of graphene mixture fluid [14]. Similar to the previously discussed method for CNT and CNF being used as a conductive filler in a polymer matrix, graphene can also be used as a conductive filler to form highly conductive biocompatible nanocomposites [34,42,43,67,83,84,85]. In some electrode designs, the graphene-filled composite acts as a component among several others in the electrode, such as a self-adhesive conductive gel to aid in skin contact and electrical conductivity [42], or as a conductive ink that can be patterned onto textiles by screen printing for EEG monitoring or other electrophysiological applications [43]. Other designs feature the graphene-filled component as a conductive layer stacked among other materials to achieve high conductivity and low skin-surface impedance while maintaining flexibility and user comfort [67,84,85]. Finally, graphene can act as a conductive filler homogeneously dispersed throughout the substrate, most similar to how CNT is frequently utilized for EEG electrodes. One such design created a conductive elastic polymer electrode by mixing graphene oxide (GO), glycerol, and polyvinyl alcohol (PVA) to create layers of varying conductive nanocomposites [83]. Another design mixes GO and silver/copper (Ag/Cu) flakes into a silicone base, which was then cured and cut into small pieces to function as EEG electrodes [34]. Graphene has been utilized in EEG electrode design the most frequently of the selected articles and is a very versatile nanomaterial, demonstrating its uses from being deposited onto the surface of a substrate in a single-atom layer to acting as conductive ink for electronic textiles and functioning as a filler for conductive polymer nanocomposites. It is worth noting that utilizing graphene in an electrode design may require more complex and less cost-effective synthesis and fabrication processes than that of CNT and CNF. However, with its advanced electrical and mechanical properties, high biocompatibility, and many forms, graphene may be a desirable option for new carbon-based nanocomposite electrode designs. A summary of the synthesis, forms, and utilization of graphene in EEG electrode design is presented in Table 3.

#### 2.1.4. Carbon Black

Carbon black (CB) is a carbon nanomaterial that consists of spherical particles on the nanometer scale that cluster together to form particle aggregates and agglomerates [86]. As with the previously discussed carbon nanomaterials, CB can be used as a filler in composite materials to modify and tune the composite’s mechanical and electrical properties, especially when high electrical conductivity is desired [87]. In the fabrication of EEG electrode design, it can act as the sole filler material or a composite filler can be created, combining CB with another filler to further enhance properties. When mixing CB into silicone to create a conductive nanocomposite electrode, it was demonstrated that the filler ratio could vary from 1.7 to 4 wt.% while maintaining high flexibility, with a maximum elongation of 840% [19]. However, this electrode design required an active amplifier circuit to successfully record EEG, and the electrodes were not as capable as the commercial standard. In another design, acetylene carbon black (AB) was mixed with a polydimethysiloxane (PDMS) elastomer to form flexible, gel-free, self-adhesive EEG electrodes [88]. When AB was mixed with PDMS at 7 wt.%, it was found to have skin-contact impedance comparable to that of commercial gel electrodes despite being a gel-free electrode. The electrodes were also capable of being used in a wireless EEG system, while most commercial EEG electrodes must be directly wired to a data acquisition or transmission device. While CB can be utilized as a filler on its own, it may be desirable to combine it with another filler material to enhance the electrical or mechanical properties of the electrode. By dispersing CB with CNF and mixing them with an SPBS polymer, they create a synergistic conductive network that can rearrange after deformations in the viscoelastic matrix, resulting in stable resistance changes [73]. The resulting viscoelastic dry electrode can achieve high conformation to the skin surface, and the CB/CNF network leads to stable EEG signal recording at the scalp. CB can also be utilized in a composite filler with other materials besides carbons, such as PEDOT:PSS, which is known to have high biocompatibility and aids in low contact impedance for electrodes [87]. Using PEDOT:PSS as a conventional filler material for polymers and combining it with CB, the PEDOT:PSS/CB polymer mixture can be molded into various electrode shapes and achieve low skin-contact impedance as well as record EEG measurements with nearly the same accuracy as commercially available wet electrodes. While CB on its own as a conductive filler material may be less capable of achieving high electrical and mechanical properties for EEG electrodes, it can be combined with other conductive materials or carbon nanomaterials to fabricate effective EEG electrodes with high signal correlation and low electrical skin-contact impedance.

#### 2.1.5. Carbon Fibers and Particles

While the most commonly utilized forms of carbon nanomaterials in EEG electrode design have been discussed, some publications have utilized carbon fibers, which are on the micro- to millimeter scale, as well as carbon particles, which were not specified by the authors to be one of the previously mentioned materials. Despite the larger size and ambiguity of these two materials, they are included as they are carbon-based composites for advanced EEG electrode design, and it is beneficial to compare alternative utilization of carbon for composite electrodes. Carbon fiber (CF) can be chopped into short strands and used as a conductive filler material for various substrates, such as medical-grade silicone for earpiece electrodes [49] or for creating a conductive silicone sponge-like electrode [50]. The CF strands have a diameter of ≈5 μm and are chopped to an average fiber length of 2–5 mm [50], allowing them to be dispersed through silicone. Single CF monofilaments can also be arranged perpendicular to the electrode surface, coated with a polymer insulating layer, and integrated into an electrode array to record EEG signal [38]. This design utilizes the CF monofilaments as the main conductive channel between the scalp and electrode rather than utilizing CF as a conductive filler. Carbon in a powdered form can be used as a conductive filler for polymers both on its own as well as with other filler materials to form a composite filler [18,51]. Another demonstrated application is using carbon to coat nylon fibers to form conductive bristles that can reach through the hair to the scalp and achieve skin contact [46].

### 2.2. Electrode Substrates

When designing a carbon-based nanocomposite EEG electrode, one of the most important considerations is the material that will act as the substrate or composite matrix. For the material to be an effective electrode substrate, it is required to have suitable mechanical and electrical properties, biocompatibility, and properties that enhance user comfort [7,8,89]. These requirements include adequate stretchability [13,80], adhesion to the skin surface [15,39,42,63], conformability to the skin shape [13,66,73], low contact impedance [41,61,67], high signal-to-noise ratio (SNR) [18,67], low cytotoxicity [62,84], and a suitable size and density [68,77]. Elastic polymers, or elastomers, are frequently selected for electrode design because many elastomers are biocompatible, cost-effective, soft, stretchable, and compatible with conductive fillers or other modifications. Polymers commonly utilized to fabricate elastic substrates include polydimethylsiloxane (PDMS) [15,39,41], styrene-ethylene-butylene-styrene (SEBS) [18,40,79], and polyvinyl alcohol (PVA) [66,83,85]. Alternatively, electrodes may be designed around textile substrates in order to integrate into wearable electronic devices [43,44], or rigid wafers to utilize lithography or to grow materials from the wafers such as silicon [81,82], and tantalum [16]. The distribution of substrate materials selected for electrode fabrication across the research reviewed in this paper is presented in Table 4. Some of the common substrate materials are discussed next in further detail.

#### 2.2.1. Elastic Polymers

Elastic polymers are a popular choice for EEG electrode substrates, as there are many commercially available elastomers that have high stretchability, conformability, and biocompatibility [90,91,92]. The skin exhibits mechanical behavior of a nonlinear stress-strain relationship and has low Young’s modulus, high toughness, and tear resistance, so it is desirable that the substrate used for an epidermal electrode has similar mechanical behavior [93,94]. The use of elastic polymers for EEG electrode fabrication includes polydimethylsiloxane (PDMS) [15,17,32,33,37,38,39,41,60,62,64,65,66,67,80,88], styrene-ethylene-butylene-styrene (SEBS) [18,36,40,79], polyvinyl alcohol (PVA) [66,78,83,85], silicone [19,34,49,50], modifications of PDMS such as viscoelasticity [73] and self-adhesion [61,63,64], polyurethane (PU) [35,38,77] and thermoplastic polyurethane (TPU) [68], poly(3,4-ethylenedioxythiophene): poly(styrene sulfonate) (PEDOT:PSS) [13,76], and ethylene propylene diene monomer (EPDM) functionalized as a polymer [51].

**Figure 5:**
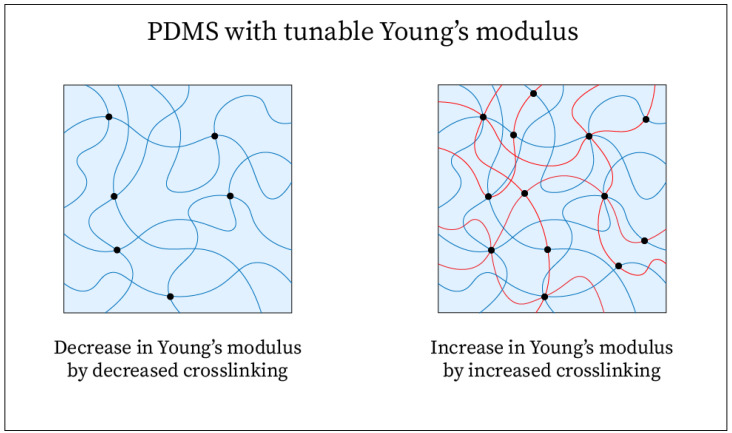
Fine-tuning the Young’s modulus of PDMS by adjusting the effect of crosslinking where an increase or decrease in crosslinking causes a corresponding change in the Young’s modulus.

Within nanocomposite electrode fabrication for electrophysiological signal recordings, PDMS is one of the most popular options for its many desirable properties. First, the elastomer has a very low Young’s modulus of 1–3 MPa [90,95,96] and can be fine-tuned by adjusting the effect of crosslinking in the polymer (Figure 5) [90,97]. It also exhibits a linear elastic strain response up to 40% strain under tensile stress and a linear elastic strain response up to 55% under compressive stress; both tensile and compressive stress/strain curves exhibit nonlinear regions beyond these strain values before failure [95]. Additionally, PDMS is known to be highly biocompatible and biostable, allowing it to be commonly used for implants in the human body [91]. With all these features and being widely available as well as cost-effective [91], PDMS makes a good choice for the EEG electrode substrate. One common way it has been used in electrode fabrication is as the matrix component of polymer-based composites with a conductive filler [15,17,33,37,38,39,41,60,62,64,65,67,88]. The PDMS/filler mixture can be poured into electrode molds to form various geometry, or it can also be spin-coated to deposit layers onto a substrate [67]. Unfilled PDMS can be used as a thin-film substrate with conductive materials deposited on its surface to form thin, skin-like electrodes [66,80]. It can also be used simply as a non-conductive base component of an electrode to hold a conductive component in place [32,88]. Modifications of PDMS may be desired depending on the electrode requirements; these include adhesive PDMS [61,63,64] and viscoelastic composites based on PDMS [73].

Another elastomer that has been used for EEG electrode fabrication is SEBS, which is valued for its strength, flexibility, and optical transparency [18,36,98]. SEBS exhibits low stiffness with a Young’s modulus of 13–43 MPa and a maximum strain of 447–500% depending on the polymer composition, and these values can be fine-tuned or increased by modifying the SEBS composition [99,100]. It also exhibits high biocompatibility, capable of being utilized as drug delivery patches [98] and able to remain on the skin surface for long periods of time without irritating [18]. Due to its high flexibility and stretchability, SEBS has been used to fabricate skin-like electrodes from thin films. The skin-like electrode can have carbon nanomaterials such as graphene, as well as carbon-based nanocomposites, deposited onto the thin-film surface to achieve conductivity, optical transparency, and high skin conformality due to the very thin nature of the materials used [36,79]. Thin-film electrodes can also be fabricated with SEBS by drop coating the polymer onto graphene patterned on a substrate, then peeling away the SEBS with the graphene adhered to the surface [40]. Aside from thin, skin-like electrodes, SEBS can be utilized in a polymer composite with conductive fillers and other thermoplastic elastomers to form a moldable mixture, similar to the previously discussed method often used to fabricate PDMS-based nanocomposite electrodes [18].

Additional elastic polymers that may be utilized in EEG electrode fabrication include PVA, PU, Silicone, PEDOT:PSS, and EPDM. While these are less commonly utilized for carbon-based nanocomposite EEG electrodes, they have been demonstrated to be viable options. PVA has been used to fabricate thin-film electrodes that conform to the skin surface [66,78,83] as well as functioned as a moldable composite matrix with a conductive filler [85]. PU has been utilized for thin-film electrodes [35,68,77] and for mechanical reinforcement of carbon fibers that penetrate through the hair to contact the scalp [38]. Silicone has also been utilized as a composite matrix with a conductive filler and formed with molds [19,49,50,67]. PEDOT:PSS is the most well-known organic conductor, being recognized for its capability to form transparent, stretchable, conductive composites for energy, electronics, and biology applications [101]. It has been functionalized as a polymer for electrode fabrication, demonstrating its capabilities in carbon-based nanocomposites as an ultra-conductive and transparent thin-film electrode substrate [13,76]. EPDM rubber has also been utilized to achieve high user comfort in the electrode design with various filler materials to optimize conductivity, flexibility, and ease of fabrication [51].

#### 2.2.2. Alternative Substrates

While elastic polymers are a widely utilized choice for the electrode substrate, some designs may choose other materials, such as textiles or wafers, as the substrate in order to fabricate more advanced morphologies or design the electrode with a very specific purpose in mind. Nylon has been utilized in several forms for carbon-based nanocomposite substrates, including nylon bristles coated with carbon to penetrate through the hair to the scalp [46], flexible nylon membranes that conform to the contours of the skin [84], and nylon textiles for integration into gel-free wearable e-textile devices for electrophysiological signal recording [44]. Cotton has also been utilized as a textile substrate for wearable e-textile devices that record electrophysiological signals [43]. Various forms of copper have been utilized as semi-flexible substrates, including flexible copper-clad laminate to form conductive channels within a structural electrode array [42] and copper foil to enhance conductivity between a carbon-based nanocomposite and the electrical wiring leading from a data recording device to the electrode itself [67]. Various types of wafers have been used to fabricate advanced electrode morphologies, particularly when microstructures or patterned graphene are desired. Tantalum wafers have been used to grow boron and nitrogen co-doped vertical graphene nanosheets via CVD, and the wafer was then inserted into a polytetrafluoroethylene (PTFE) electrode holder to record EEG signal [16]. Titanium wafers have been used to grow TiO_2_ nanotubes via anodization to enhance the electrode’s conductivity, and few-layer graphene could then be deposited onto the nanotubes via CVD [32]. Silicon (Si) and silicon carbide (SiC) wafers have been utilized for photolithography and photo etching to form micropillar and micropyramid arrays to enhance signal quality when recording electrophysiological signal from hairy skin [14,81]. SiC wafers have also been utilized for growing epitaxial graphene on the wafer surface via the catalytic alloy approach for use in an EEG electrode [82,102,103]. While these wafer-based substrates may allow for more advanced and complex electrode designs, they may reduce user comfort due to the rigid design, particularly when recording EEG signals for extended periods of time.

### 2.3. Surface Contact Structures

**Figure 6:**
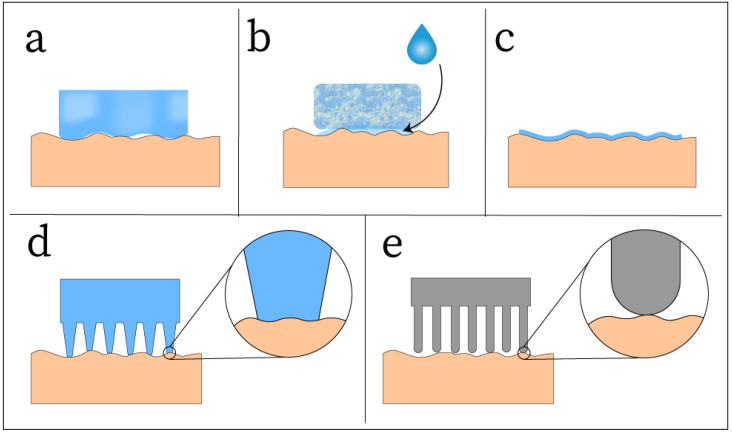
Illustrations of various skin-surface contact methods in electrode design: (**a**) ultra-soft surface with low elastic modulus to achieve high contact with the skin surface; (**b**) sponge-like design to absorb sweat from the skin; (**c**) ultrathin film to achieve conformal contact with the skin surface; (**d**) soft microstructures to achieve contact through hair on the scalp; (**e**) traditional rigid dry electrodes with pronged design to achieve contact through hair on the scalp.

One of the main challenges in noninvasive EEG electrode design is the formation of a stable interface between the electrode and the microscopic contours of the scalp, which is commonly covered with hairs [15,104]. Fabricating structures on the electrode surface or utilizing highly conformable materials to achieve higher skin contact can greatly reduce the skin-contact impedance and achieve a more stable signal. These conformal mechanisms increase the electrode’s contact surface area and reduce the insulating barrier of hair or air that may be present between the scalp and the electrode. A simple way to increase conformal contact is by fabricating the electrode with a very soft material (Figure 6a). Soft, flexible polymer nanocomposite electrodes can be capable of conforming to the contours of the skin to achieve stable contact, as demonstrated in many electrodes designs [17,19,37,39,41,49,50,60,61,62,63,64,65,67,85,88]. This is attributed to the low Young’s modulus of the polymers used in electrode fabrication, as a lower Young’s modulus will result in better conformality to the rough skin surface [11,94]. Additionally, an electrode made of a viscoelastic carbon-based nanocomposite can take this principle further, being able to flow and fill into the skin surface, achieving very high skin contact [73].

Another mechanism that can be used to enhance surface contact is designing the electrode with a sweat absorption capacity (Figure 6b) [16,82]. As sweat is secreted from the skin, it can be absorbed by the electrode, which can improve the electrode/skin interface by softening the electrode and enhancing electrical conductivity [105]. This mechanism naturally mimics that of wet electrodes, which use a conductive gel to hydrate the skin and ensure a low-impedance conductive channel to record EEG signal [106]. Some polymers, such as PDMS, are hydrophobic and will not respond well to the presence of sweat [90]. However, graphene with many atomic-level defects can absorb sweat due to the formation of C-OH bonds between the sweat and graphene grain boundaries, making graphene suitable for sweat-absorbing electrodes [16,82].

**Figure 7:**
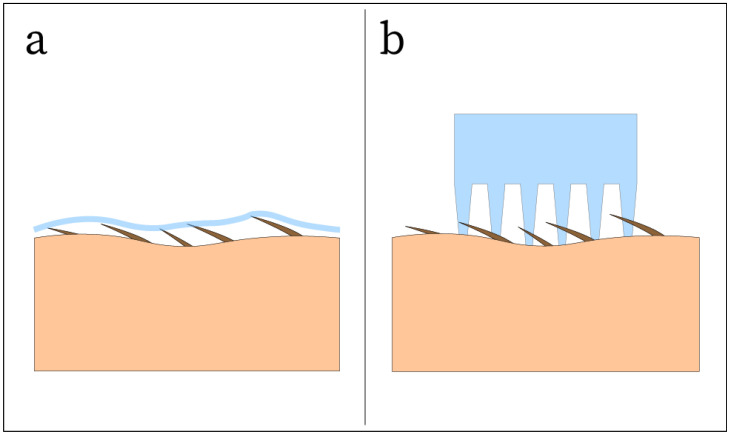
Illustrations of (**a**) thin-film electrode’s weakened of surface contact when hair is dense on the skin; (**b**) pin-like electrode able to reach through dense hair and contact the skin.

Skin-like thin film electrodes have been demonstrated to be highly conformable to the contours of the skin due to the nature of their thin design [13,35,36,39,40,66,68,76,77,78,79,80,83,84]. These films can be ultrathin, soft, and lightweight, allowing them to be mounted to the skin by van der Waals forces alone, requiring no external adhesives [107,108]. These thin films can range from as thin as 129 nm [76] up to 500 μm [39]. As the film becomes thinner, it will become more deformable and more easily conform to the skin surface, allowing for very high conformality (Figure 6c). In a laser-scribed graphene(LSG)/PU thin film electrode with thickness of 26.2 ± 6.9 μm, the film was placed on the tip of a finger, and clear fingerprint morphology was visible in the LSG/PU film [77]. Numerical studies have also been performed, indicating that a thin-film thickness smaller than 27.5 μm can maintain conformal contact with hairless skin [107]. This suggests that films of equal or less thickness should easily be able to conform to a skin surface when no hair is present. However, this is not the scenario that most EEG electrodes are used in, causing thin-film electrodes to be ineffective in the presence of hair (Figure 7a). To mitigate the challenge of the presence of hair in EEG recording areas, structures such as pins can be fabricated on the electrode surface to reach through the hair and make contact with the scalp (Figure 7b).

A common method for designing dry EEG electrodes to achieve skin contact on the hairy scalp is fabricating micro- or macrostructures on the electrode surface. One approach is with spiky contact, where the electrode contains an array of pins, spikes, or prongs which may be in the scale of nanometers, micrometers, and millimeters (Figure 6d) [24]. These structures exist in both invasive and noninvasive implementations, where the invasive designs use microneedle arrays to penetrate the stratum corneum, or the outermost layer of the epidermis [109,110,111]. The noninvasive designs use arrays of prongs, bristles, or other geometry to contact the skin without damaging it. The reviewed carbon-based nanocomposite designs only included noninvasive approaches, likely because one of the objectives of advanced EEG electrode design is to optimize user comfort. The larger, millimeter-scale structures are typically pins that extrude from the surface of the electrode and can vary in length and diameter depending on the desired surface contact area and density of hair on the scalp. The reported lengths and diameters of the pins fabricated with carbon-based nanocomposites ranged from 2 to 8 mm and 1.5 to 3 mm, respectively [18,33,51,87]. While pins can reach the scalp through hair, they still need a mechanism to conform to the microscopically rough skin surface. This is where microstructures may be utilized, as these structures are fabricated on the micron scale and can take on a variety of shapes and designs [15,67]. Variations on the millimeter-scale pin design have been investigated, such as fabricating conical suction-cup-like microstructures on the end of each pin that use suction force to adhere to the scalp and improve signal quality [15]. Aside from pin designs, microstructures may be fabricated on the surface of the electrode rather than in the form of pins. One such design fabricated Ag microclaws of cylindrical shape with a diameter of ≈600 μm, which extruded from the surface of the electrode [67]. The claws, in combination with a CNT-GO-PDMS network, created a dual-mode electron transfer, resulting in high SNR. Another example is a matrix of micro-pyramids, each with a square area of 16 × 16 μm and a height of 80 μm, fabricated by photoetching on a SiO_2_ wafer [14]. These microstructures were used as a mold for graphene-based electrodes, allowing for the fabrication of graphene electrodes with matrix micropyramid structure. A third example is the usage of photolithography to fabricate micropillars on an Si substrate with a height of 10 μm and different shapes in the range of 1–5 mm^2^, which could then be used to grow epitaxial graphene and function as an electrode [81]. Each of these surface contact methods has advantages and disadvantages in their effectiveness versus cost to manufacture, but an ideal surface contact method may involve the combination of one or more of these methods. Commercially available dry Ag/AgCl electrodes commonly utilize a pin-shaped design to contact the scalp through a layer of hair, but the rigid material of the electrode does not conform to the skin and may cause discomfort to the user (Figure 6e). Therefore, the combination of soft, conductive carbon-based nanocomposites and pin-shaped designs may lead to an advanced dry electrode that can greatly reduce skin-contact electrical impedance by allowing the pins to conform to the skin at the micrometer scale (Figure 6d).

## 3. Electrode Properties

**Figure 8:**
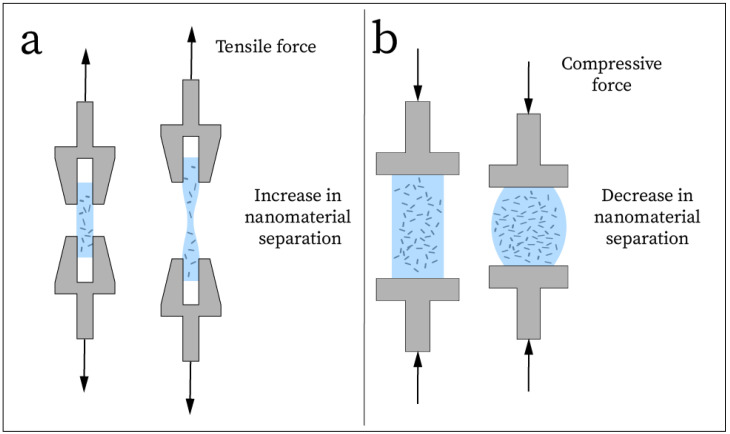
Example of testing the mechanical properties of a polymer nanocomposite via (**a**) tensile testing and (**b**) compression testing.

To develop a carbon-based nanocomposite electrode for EEG recording, optimization of the material properties must be considered. The mechanical properties include stretchability, adhesion, conformability, and thickness, while the electrical properties include low impedance, high conductivity, low resistance, and high signal-to-noise ratio (SNR) [89]. Another property that must be considered for many carbon-based nanocomposite sensors is how the material properties change in response to strain [48]. This property is known as a piezoresistance effect present in conductive nanofiller-based materials due to the change in distance between filler materials (Figure 8) under external load [10,48,112]. This piezoresistivity can cause changes in recorded signals depending on the external load applied to the electrode, so it is necessary to test and record this property for flexible nanocomposite electrodes. To validate the suitability of advanced electrodes, their material properties will be compared to that of the commonly used, medical-grade wet Ag/AgCl electrode, as well as commercially available dry electrodes that may be used in research environments.

### 3.1. Mechanical Properties

In order for an electrode to be most suitable for electrophysiological recording, it must have mechanical properties that are similar to that of the human skin [13,93,94]. The epidermal layer of the human skin has been characterized to have a Young’s modulus of 130 kPa, and it can experience strain of up to 30% in daily activities [107,108]. Therefore, the electrode’s maximum elastic strain, Young’s modulus, hardness, and adhesion force are indicators of how effective and comfortable it will be for the user. Long-term stability over repeated use cycles also must be measured, and this can be done through loading-unloading stress cycles to measure hysteresis and changes in other material properties [41,62,68].

First, the maximum elastic strain of the electrode must be beyond that of human skin so that it can stretch with the skin without experiencing plastic deformation. Additionally, the material should be able to withstand this strain without experiencing irreversible changes in mechanical or electrical properties. Nanocomposites based on carbon-filled PDMS elastomers can easily attain this level of stretchability, with maximum strains ranging from 40 to 120% depending on the ratio of PDMS base to curing agent and the filler volume [15,17,33,41,62,65]. Nanocomposites based on SEBS can also achieve this stretchability, reaching 105.68% strain when fabricated with bulk conductive fillers [18]. Thin-film electrodes can achieve higher maximum strain, ranging from 40 to >300% strain when fabricated with SEBS [13,36,40]. Silicone thin-film was demonstrated to reach 840% strain [19], and TPU thin-film was demonstrated to have elastic recovery at 30% strain and a maximum of 4000% strain [68]. Clearly, carbon-based polymer nanocomposites are well suited for epidermal electrodes in terms of elastic strain.

Next, the Young’s modulus of the electrode material should be similar to that of human skin (≈130 kPa) [107,108] to be most comfortable for the user [93,94]. Based on the reported values, PDMS nanocomposites tend to have higher moduli than SEBS nanocomposites, typically being in the range of 1–4 MPa [37,38,40,41,62,65]. However, there are outliers where the modulus was tuned to be nearer to that of human skin by adjusting the PDMS base to filler ratio, such as PDMS nanocomposites with moduli of 130 kPa [63] and 16 kPa [61]. SEBS nanocomposites tended to have lower reported moduli, being in the range of 100–760 kPa [13,18,73]. Nanocomposites with other polymers demonstrated similarly low moduli, including a TPU nanocomposite with a modulus of 3.36 MPa [68] and EPDM nanocomposite with a modulus ranging from 30 to 170 MPa [51]. While the majority of the reported Young’s modulus values are higher than that of human skin, they are significantly lower than that of commercially available dry electrodes, which are commonly fabricated from injection-molded acrylonitrile butadiene styrene (ABS) with a Young’s modulus in the range of 1.5–1.7 GPa [113].

Adhesion force is another property that must be considered, and it is defined as the force required to remove the electrode from the skin surface [114,115]. The adhesive force can be provided by the material itself or by an external adhesive applied to the electrode or skin surface. The adhesion force ranged from 0.23 to 10.51 N/cm^2^ for thin-film electrodes [64,66,68,80], and from 0.2–3.71 N/cm^2^ for bulk filler composite electrodes [39,40,61,63]. These values include ultrathin film adhesion due to van der Waals forces, self-adhesive polymers, and adhesive pastes. Ideally, the electrode should have a self-adhesive property to simplify usage and reduce setup time, as van der Waals forces are not suitable for thicker electrodes, and adhesive pastes are inconvenient for the user. Most reviewed electrode designs displayed an adhesion force around 1.5 N/cm^2^, so this value should be considered to be the minimum when designing a self-adhesive electrode.

The life cycle and long-term stability of flexible nanocomposite electrodes must also be evaluated through cyclic loading-unloading tests to record any possible hysteresis or changes in material properties [116]. When the electrode undergoes stress-strain testing, both open and closed hysteresis loops have been documented during the first stress-strain cycle [17,41,62,68]. Increasing filler content typically corresponds with an increase in mechanical hysteresis, which can lead to plastic deformation occurring during loading and unloading cycles [17]. If the nanocomposite exhibits a closed hysteresis loop within strain values that exceed that of the human skin, such as 0–50%, then it suggests good elasticity [41]. The hysteresis loop may also stabilize over a few number of cycles, such as ten [68] to 100 [15] consecutive cycles, resulting in a stable material after the “break-in” period. In this case, the electrode should undergo the necessary number of loading-unloading cycles to stabilize before testing its electrical properties to achieve accurate results. Once the initial hysteresis loop has been characterized, long-term stability should be tested with cyclic loading and unloading of the electrode to examine relative change in resistance over the electrode’s life cycle. The majority of carbon-based nanocomposite electrodes demonstrated good stability, with very low relative change in resistance over a varying amount of loading-unloading cycles, including 100–500 cycles [15,40,73], 1000–2000 cycles [14,35,36,66,77], and 5000–10,000 cycles [41,43,62,83]. These loading cycles most commonly consisted of tensile stress ranging from 30 to 50% strain. However, there are some exceptions to this stability of carbon nanocomposite electrodes, such as an increase in electrical impedance after only five cycles [79] and a relative change in resistance of 10.84% after 1500 cycles [68]. The long-term stability over thousands of cycles demonstrated by many carbon-based nanocomposite electrode designs is not found in commercially available wet or dry electrodes. Wet electrodes dry out in a matter of hours, greatly increasing electrical impedance and reducing adhesion force, and the conductive plating on dry electrodes is worn down over repeated uses, resulting in decreased signal quality.

### 3.2. Electrical Properties

EEG signals generated by the human brain have very low voltage and frequencies, typically around 10–100 μV and 1–100 Hz [117,118,119]. As a result, these signals are more difficult to measure noninvasively than other electrophysiological signals such as electrocardiogram and electromyogram [119]. EEG electrodes, then, must have advanced electrical characteristics, including low skin-contact impedance, low resistance, and high conductivity [28,89]. In recent advancements, high-quality EEG signal with high SNR acquired from noninvasive, dry EEG electrodes has been attributed to the aforementioned electrical qualities [35,40,76]. Unfortunately, designing a successful electrode may not be as simple as maximizing or minimizing these properties, as there is not yet a universally accepted benchmark for EEG signal acquisition that new electrodes can be verified. It is generally understood that low skin-contact impedance and high SNR lead to better signal acquisition, but the skin-contact impedance is a function of not only the electrode’s conductivity and resistance but also its skin-surface conformability. Additionally, many different methods are used to measure the electrode’s conductivity, impedance, and other qualities; there is no set standard. This can clearly be seen when examining the reported skin-contact impedance values for the commercial wet Ag/AgCl electrode. Many articles state that the commercial wet Ag/AgCl electrode should have an impedance of ∼10 kΩ, but the measured impedance values for this type of electrode have high variability, ranging from 0.1 kΩ [87] to 25 kΩ [66] at 1000 Hz and 0.5 kΩ [87] to 300 kΩ [65] in the low-frequency range of 4–10 Hz, with many values in between [40,66,67]. The measured conductivity of the carbon-based nanocomposite electrodes also displays wide variation, ranging from 0.33 S/m [44] to 4.142 × 10^5^ S/m [13], but these designs all claim to be effective for EEG signal acquisition. Given the wide variation of electrical properties (e.g., conductivity) of electrodes, there should be a different figure of merit for characterizing the performance of EEG electrodes. Instead, EEG signal acquisition and signal characteristics such as SNR could be used to benchmark the electrodes. This would give a clear, real-world example of how effective the electrodes truly are. The reported values for electrode resistance, impedance, and conductivity are presented in Table 5 to provide a clear comparison of the current literature, but these values should not be used as the sole way to judge the electrode’s effectiveness.

### 3.3. Strain Response

In many carbon-based nanocomposites, particularly those that use carbon nanoparticles as a conductive filler, it has been well documented that a piezoresistive effect is generated as the nanocomposite undergoes strain [10,48,112,120,121,122]. The conductivity of the carbon-filled nanocomposite changes as the strain increases or decreases due to the formation and destruction of conductive networks throughout the composite [48,112]. Thus, for soft, dry carbon-based nanocomposite EEG electrodes, this piezoresistance effect may be present as the electrode is compressed or stretched on the skin surface, causing changes in signal quality during recording. Much of the reviewed literature discusses this effect, describing the relative change in resistance while the electrode undergoes tensile, compressive, and bending strain, which may be useful in predicting how new carbon-based nanocomposite electrode designs will behave under stress.

**Table 5:** Summary of the reported electrical properties of various carbon-based nanocomposite electrode designs.

Electrode Type	Resistance or Resistivity ^a^	Skin-Contact Impedance ^b^	Conductivity	Reference
Ag/CNT/PDMS Composite	216–663 Ω/sq	-	0.75–3.70 S/m	[41]
CNT/PDMS Thin-film	-	800 kΩ at 10 Hz	10–100 S/m	[39]
GPG Thin-film	20.8 Ω/sq	200 kΩ at 10 Hz	2850–3727 S/m	[76]
SLPP Thin-film	90–180 Ω/sq	14.39 kΩ at 10 Hz	-	[40]
CMSA Composite	1387 Ω/sq	275 kΩ at 20 Hz	-	[15]
TCRE Composite	-	∼10 kΩ at 10 Hz	-	[42]
PEDOT:PSS Composite	0.2 Ω·m	10 kΩ·cm^2^ at 10 Hz	-	[87]
ACNT Thin-film	250–2000 Ω/sq	500 kΩ at 50 Hz	1.2 × 10^4^ S/m	[66]
Ag/CNT-GO-PDMS Composite	9.66–14.3 kΩ	∼15 kΩ at 8–13 Hz	-	[67]
FLG/TiO_2_ Wafer	9.2–19.0 kΩ	-	-	[32]
CNT/aPDMS Composite	-	309.8 kΩ at 10 Hz	-	[61]
AB/PDMS Composite	10–100 kΩ	271 kΩ at 100 Hz	-	[88]
SPRABE Thin-film	108 Ω/sq	100 kΩ at 10 Hz	-	[68]
EG/SiC Wafer	-	155–325 kΩ at 50 Hz	-	[81]
BVNG Electrode	∼15 kΩ	∼5 kΩ at 10 Hz	-	[16]
LSG/PU Thin-film	30–70 Ω/sq	∼2.5 × 10^4^ kΩ at 50 Hz	-	[77]
Cu-TiO_2_-CNT@PDMS	10.9–12.62 kΩ	<5 kΩ at 10 Hz	-	[33]
PTG Thin-film	24–170 Ω/sq	32 kΩ at 100 Hz	2850–4142 S/m	[13]
EARtrodes composite	-	<60 kΩ at 10 Hz	-	[49]
rGO Textile	14 kΩ/sq	∼59 kΩ at 50 Hz	∼0.33 S/m	[44]
EG Composite	0.1–0.2 Ω·m	128 kΩ at 10 Hz	-	[82]
GFG Composite	150–275 Ω/sq	2 × 10^4^ kΩ at 10 Hz	-	[79]
AgNW-GES Thin-film	700 Ω	100 kΩ at 10 Hz	-	[35]
rGO Thin-film	-	600 kΩ at 20 Hz	-	[80]
TRGO/NM Thin-film	∼40 Ω/sq	∼20 kΩ at 4 Hz	-	[84]
GEMMPS Wafer	30 Ω	∼250 kΩ at 10 Hz	-	[14]
CNT/PDMS Composite	0.1–100 kΩ	0.005–1 kΩ at 10 Hz	-	[17]
AgNW/CNT Composite	-	∼200 kΩ at 10 Hz	-	[37]
CF Composite	-	∼200 kΩ at 20 Hz	1–18 S/m	[50]
CF Bristles	-	40–60 kΩ at 10 Hz	250–1000 S/m	[38]

^a^ Units of Ω/sq indicate sheet resistance [123], units of Ω or kΩ indicate bulk resistance, and units of Ω·m indicate resistivity. ^b^ Values are reported for the lowest frequency provided in each respective research, as EEG signals are generally very low frequency. The reported kΩ·cm^2^ corresponds to a normalized impedance that compensates for true exposed electrode surface area.

In general, electrodes that used a conductive polymer nanocomposite as the bulk of the electrode experienced a less drastic change in resistance due to strain than the electrodes based on thin-film designs. CNT-filled polymer nanocomposite electrodes reported a relative change in resistance (ΔR/R_0_) ranging from less than 2% up to 15% when undergoing a tensile strain of 40 to 50% [15,39,41,62]. Under compression, [15] reported a relative resistance change of <4% for their CNT-filled polymer nanocomposite at 40% compression, while in [17], it was reported that the conductivity of the CNF-filled polymer nanocomposite increases under compression, but the dependence can be eliminated by increasing filler loading. The behavior under compression of these electrodes can be explained by both rotations of nanofibers as well as a decrease in interfiber distance [10]. As the interfiber distance decreases, the amount of conductive pathways through the filler matrix rapidly increases, increasing the electrical conductivity of the material. The reported thin-film electrodes used layers of graphene or CNT to provide the conductive network, and these electrodes tended to experience much more drastic resistance changes due to tensile strain. Their strain responses included ∼150% relative change due to 35% strain [76], 100–200% change due to 50% strain [68], ∼1000% change due to 50% strain [77], 300% change due to 40% strain [13], ∼35% change due to 50% strain [84], and 160% change due to 5% strain [35]. Some thin-film electrodes demonstrated less dramatic resistance change comparable to the filled nanocomposite electrodes, including a 3.75% change due to 30% strain [40], <5% change due to 70% strain [66], and ∼15% change due to 30% strain [36]. Since the thin-film electrode geometry is affected much more by strain than a larger, filled composite-based electrode, it is reasonable to expect that thin-film electrodes will perform worse under strain. However, for the application of EEG recording, the electrodes do not experience high tensile strain. Rather, they may experience compression if external force is applied to press down onto the scalp.

### 3.4. Signal-to-Noise Ratio

One of the most commonly utilized methods to determine an EEG electrode’s signal acquisition effectiveness is by measuring the electrode’s signal-to-noise ratio (SNR). The SNR is measured in decibels (dB) and represents the ratio of the desired signal to baseline noise [41]. There are several equations that have been used to calculate SNR. First, one method is represented by Equation (1),

(1) $$SNR=20\times log\left(\frac{A_{signal}}{A_{noise}}\right)$$

where A represents the root-mean-square (RMS) amplitude values of the signal and noise, respectively [40,76]. Another similar equation has been used to calculate SNR that replaces the RMS component with a fast Fourier transform (FFT) shown in Equation (2),

(2) $$SNR=20\times log\left(\frac{\gamma (f)}{\gamma (f-0.125)+\gamma (f+0.125)}\right)$$

where γ(*f*) (=amplitude (μV)/$\sqrt{f}$ (Hz)) is the amplitude spectrum calculated from the FFT [16,32,33,67]. Other methods utilized to calculate the SNR include an RMS ratio without the logarithmic component depicted in Equation (3),

(3) $$SNR={\left(\frac{A_{Closed}}{A_{Open}}\right)}^{2}$$

where A represents the RMS amplitude for closed and open eyes [19], and another method used a ratio of the power density of target frequencies versus *N* neighboring frequencies, expressed in Equation (4),

(4) $$SNR=\frac{P(f\times N)}{\sum_{k=1}^{N/2}P\left(f+k\times r\right)+P\left(f-k\times r\right)}$$

where P(*f*) is the estimated power amplitude at a frequency *f*, *r* is the frequency resolution, which was set to 0.1, and N is the number of neighboring frequencies, which was set to 20 [34]. Another way that SNR has been represented is the ratio of the signal’s power spectral density (PSD) to the PSD of the background noise, as shown in Equation (5),

(5) $$SNR=10\times log\left(\frac{\frac{1}{N}\sum_{i=1}^{N}\left(PSDup\right)}{\frac{1}{N^{\prime }}\sum_{j=1}^{N^{\prime }}\left(PSDdown\right)}\right)$$

where *N* and $N^{\prime }$ are the signal and noise considered at the number of up and down states and PSDup and PSDdown are the PSD of the signal and noise, respectively [81]. SNR can be interpreted as the ratio of the desired target signal to the noise present in the recorded signal. A higher SNR indicates better signal quality because the electrode is recording a smaller amount of noise or a greater amount of the target signal. A summary of the reported SNR values for each of the reviewed carbon-based nanocomposite electrodes is presented in Table 6, including the SNR that was measured and reported in each study for the commercial wet Ag/AgCl electrode that is commonly used as the target for new electrode developments.

## 4. Electrode Characterization

While material properties may be a strong indicator of how each electrode material compares to one another, they cannot be the only metric used to assess the electrode’s EEG acquisition suitability. Each electrode design has certain characteristics that make it more or less suitable to record EEG from the human scalp. These include biocompatibility, user comfort, and EEG acquisition performance [89]. High biocompatibility has been regarded as the most significant characteristic of skin-contacting electronic devices to ensure that they are safe for the user [89,124]. User comfort is less critical but still a necessity if the electrode is to be used for long recording sessions, as an uncomfortable electrode is very undesirable for the user. EEG performance does not yet have a standardized benchmark as the recording conditions have extremely high variation, but many researchers record known EEG phenomena to characterize the electrode performance such as alpha wave rhythms [41], P300 responses [39], sleep rhythms [76], and oddball paradigms [18]. Each of these will be discussed in the following sections to explore how these characteristics can be optimized.

### 4.1. Biocompatibility

Biocompatibility, based on in vivo and in vitro biotoxicity, is an extremely important characteristic for on-skin applications such as epidermal electrodes [124]. The conductive material must be nontoxic to the epidermis, and it also must allow reasonable ventilation and thermal comfort to prevent skin lesions and inflammation [124]. Many of the materials selected for electrode design are well documented to be biocompatible, such as PDMS [39,41]. However, for novel materials, it may be beneficial to evaluate the biotoxicity before placing it onto the skin surface. To characterize the biocompatibility of a material before placing it on human skin, a cytotoxicity test may be utilized to evaluate how the skin cells will respond to the foreign material. One method of measuring biocompatibility is by performing in vitro cell viability tests with fibroblast cells, such as mouse fibroblasts [73]. After the cells have been exposed to the novel material for a set duration, the cell viability can be analyzed using a cell counting kit to determine the number of living and dead cells. The in vitro cell viability test can also be performed with human fibroblast cells, and the same live/dead cell viability test can be performed to evaluate the biocompatibility of the novel material [36,61,63,65]. While fibroblast cells are commonly used for these tests, another cell that may be selected is the HaCaT cell, which is a human keratinocyte. These cells have been selected because they are the outermost cells of the human epidermis layer, making the biocompatibility test more aligned with how the electrode would be used [62,84]. The cell viability tests across the literature tended to have the result that 87–94% of cells were living after a duration of 1–7 days, indicating that the reviewed novel carbon-based nanocomposite materials are nontoxic to cells.

In addition to in vitro cytotoxicity tests, another biocompatibility test that can be performed is an on-skin test, where the electrode material is placed on the skin for a set duration [41]. After the material is removed, the skin surface is examined to observe changes such as irritation, redness, or other damages. In the reviewed literature, none of the carbon-based nanocomposite electrodes that were tested in this way caused skin irritation after being exposed to the skin for a duration of 8–24 h [38,41,42,60,61,66]. Electrodes with rigid or soft prong-shaped geometry may leave depressions on the skin surface, but these will fade away within minutes, returning the skin to its original state [38]. It is worth noting that an on-skin test should not be performed before an in vitro cytotoxicity test if the biocompatibility of the material is in question, as this would be a risk to the electrode wearer. The majority of literature that performed an on-skin test either performed a cytotoxicity test first or utilized materials that have been well documented to be biocompatible, such as PDMS. If the materials are known to be safe for skin contact, then an on-skin test may be appropriate.

### 4.2. EEG Performance

To study the suitability of novel electrodes for EEG signal acquisition, they must be placed on the scalp and used to record real data. The question is, then, what is the optimal paradigm to measure the performance of an electrode for EEG acquisition? This performance measurement is difficult to characterize in a way that can be universally compared to other novel electrodes due to the high variation in recording conditions and hardware. Instead, various EEG paradigms exist that can be repeated by different subjects with similar signal results, and methods such as signal correlation, power spectral density (PSD), and fast Fourier transform (FFT) can be used to quantify the performance of the electrode. There are several common EEG paradigms used to evaluate performance:

- Alpha Rhythm [15,16,17,19,35,36,40,41,42,44,62,66,68,78,79,80,84,88]
- P300 Event-related Potential (ERP) [18,39,46,49]
- N100 ERP [61,63,64,65]
- Sleep Rhythms [13,43,76]
- Visual Evoked Potential (VEP) [77]
- Auditory Evoked Potential (AEP) [33,38,64,65,73]
- Steady-state Visual Evoked Potential (SSVEP) [16,32,33,34,38,46,60,61,63,65,67,81,87]
- Auditory Steady-state Response (ASSR) [49,60,64,65,67,87]
- Signal changes due to mental state [37]

The goal with each of these paradigms is to record a phenomena that is known to occur in EEG signal when the subject experiences a certain stimulus. For example, alpha rhythms in the range of 8–13 Hz are expected to appear when the subject’s eyes are closed, and the subject is relaxed [41]. A signal processing method such as FFT can then be applied to the data, and the amplitude of the alpha signal can provide information on the characteristics of the electrode. Event-related potential is another known phenomenon that occurs due to certain stimuli, allowing it to be used for electrode performance testing. The P300 component is an ERP characterized by a positive signal deflection that occurs approximately 300 ms after a “rare” or unexpected stimulus is presented to a subject [125]. Similarly, the N100 component is a VEP characterized by a negative signal deflection that occurs approximately 100 ms after an unexpected stimulus [126]. VEP and AEP are two more forms of ERP, where a deflection in signal is caused by a visual or auditory stimulus. SSVEP is the process of displaying a steady, repetitive visual stimulus, such as an image flickering at a certain frequency. As the subject views the stimulus, an EEG response of the same frequency is induced [127]. ASSR is similar to SSVEP in that it utilizes a steady frequency to induce a response in the EEG signal, but it uses auditory stimulus rather than visual [128]. Lastly, changes in emotion or mental state, such as alertness and focus, can be classified in EEG signal, so it has also been used as a metric for EEG performance [37]. While these paradigms are beneficial for testing an individual novel electrode and comparing it to a commercial electrode, they are not effective when it comes to benchmarking electrode performance. High variability exists within not only the EEG paradigms but also the individual users and recording environments. When so many paradigms, hardware, environments, and individuals exist in research, it is unrealistic to compare one novel electrode performance to another, as they would not be compared by the same performance metrics. Instead, some of the reviewed electrode performances will be discussed to gain an understanding of how the electrodes perform individually without comparing their performance to each other.

The most common evaluation of electrode performance is testing if the electrode can detect alpha rhythms. The alpha rhythm can be visualized by recording the EEG signal over a duration, such as 1 min with the user’s eyes closed, then applying PSD or FFT to the signal to reveal a peak frequency in the band of 8–13 Hz [40,41]. Basic EEG signal characteristics such as alpha rhythm and eye blink artifacts provide simple ways to validate an electrode’s performance. Event-related potentials, such as P300, N100, VEP, AEP, SSVEP, and ASSR, require an experiment and stimulus for the user, requiring more setup than frequency rhythms and muscle artifacts. However, ERPs may provide a better indication of electrode performance as they tend to be more difficult to record. This makes clear recording of ERPs a better indicator of electrode effectiveness than simply detecting muscle artifacts. SSVEP, the most commonly utilized form of visual evoked potential, typically involves a graphical interface presented to the user that displays cues, such as letters or commands, which flicker at specific frequencies. By focusing one’s vision on a flickering cue, SSVEP is induced in the EEG data corresponding to the frequency being focused on [127]. SSVEP can be used to quantify electrode performance by applying PSD to the signal and inspecting the frequency spectra to compare the intensity of the peaks at the SSVEP frequencies between the novel electrode and a baseline electrode [32,34,38,63,65,87]. FFT can be used in the same way to perform an analysis of frequency spectra [60,61]. SSVEP can also be used in machine learning classification tasks to automate the inspection process, and the paradigm has yielded a classification accuracy of 89.6–94% [67,81].

The second most commonly utilized ERP is auditory evoked potentials, particularly auditory steady-state response. ASSR is the most widely utilized auditory stimulus paradigm [60], and it is the audio-based equivalent of SSVEP; the paradigm is invoked by the periodic modulation of an auditory tone at specific frequencies [128]. The advantages of ASSR include simple stimuli delivery by headphones or speakers, and the user is not required to direct their head or gaze toward specific targets [128]. The latter could also be seen as a disadvantage, as it would be difficult for the user to select commands in a BCI system with auditory stimulus. Therefore, ASSR may be more suited for electrode testing and machine learning validation rather than a real-time BCI implementation. It could, however, be used to complement SSVEP, as demonstrated in the SSVEP-multiple ASSR paradigm [67]. To examine electrode performance, ASSR can be induced, and PSD applied to the EEG data, then the power spectrum can be visually inspected to compare the peak intensities of the novel electrode versus the commercial electrode [49,60,65,87]. Another AEP paradigm that may be utilized is the use of auditory stimulation where the stimulus is pure tone at a specific frequency [33]. The frequencies of these pure tones induce peaks in the frequency-amplitude spectra that correspond to the tone frequencies, and the peak intensity can be compared to a baseline commercial electrode. Each ERP tends to follow a similar pattern, where a stimulus is presented to the user, and an effect is induced in the EEG signal. PSD, FFT, or similar can be applied, then the frequency analysis can be visually inspected, or the results can be used as input to train a machine learning algorithm to create a real-time BCI.

## 5. Discussion and Future Opportunities

Several recurring themes within carbon-based nanocomposite electrode designs for non-invasive EEG have been revealed, which are worth noting for further research. First, there is a variety of carbon nanomaterials available (CNT, CNF, Graphene, etc.), but the literature lacks sufficient supporting evidence as to which materials show clear advantages over the others in EEG electrode performance. Perhaps this is due to the lack of a generalized and robust method to characterize their performance. However, the literature seems to show advantages related to cost, manufacturability, availability, and electrode form. CNF appears to be more cost-effective than CNT, but CNT can have higher mechanical and electrical properties than CNF. Graphene is more applicable for thin-film electrode designs, while CNT and CNF are more effective for bulk filler designs. To make the appropriate selection, one must consider the electrode’s shape, intended use, and cost. Each form of nanocarbon has been proven to be effective, but some are more suited than others for different nanocomposite designs. One area that could benefit from further investigation is related to the understanding of the combined effects of the alignment and loading ratio of the carbon nanomaterial on the enhancement of the electrode’s electrical properties. Of the reviewed literature, only one EEG electrode utilized aligned CNT (ACNT) sheets. The alignment of CNT demonstrated high performance compared to randomly oriented CNT electrodes, and the ACNT sheets are known for their high electrical conductivity and excellent mechanical properties [66]. Orientation of CNT within polymers has been demonstrated to enhance electrical properties in other conductive polymers [129,130,131], so it has the potential for increasing the performance of EEG electrodes.

In addition, electrode surfaces should include some form of combined contact structure and microstructure (or hierarchical structure) if it is intended to record EEG from the scalp. In most scenarios, there will be hair present on many of the scalp recording locations, and the microstructures allow the electrode to reach through the hair and make conformal skin contact (in contrast to a flat electrode). Mechanically, the electrode must be rigid enough to penetrate through the hair while soft enough to make conformal contact and sufficiently comfortable to the user. To achieve this, it could be beneficial to create a hybrid layered composite/nanocomposite material with gradient properties such as low-to-high Young’s modulus. For instance, the low-modulus material could contact the skin, providing comfort and conformability, while the high-modulus material could comprise the bulk of the electrode, giving it sufficient rigidity to penetrate through the hair on the scalp. Electrically, it is desirable for the electrode to have low skin-contact impedance, low resistance, and high conductivity. However, it is somewhat indeterminate how these properties should be optimized. There is a high variation of these properties within the reviewed literature, yet most of the novel (yet low technological readiness level) electrode designs demonstrated better EEG performance than commercially available electrodes. Very importantly, the nonstandardized methods exhibited throughout the literature make comparison of performance very difficult at present. There seems to be a high variation of subjects used and testing methods, making it difficult to determine a specific property or combination of properties that definitively makes the electrode have higher EEG performance. This latter topic could provide a feasible path toward creating standard methods that could prove beneficial to the organized advancement of EEG electrodes.

Furthermore, while it is known that the maximization of the SNR is desirable (as this correlates to the high-quality signal recorded), the current employment of this figure of merit for performance may be shortsighted. One challenge in using SNR as a metric to compare the EEG performance of novel electrodes is that the equations used to calculate SNR, as well as the methods used to record signals, have high variability. Ideally, the equation and recording methods should be standardized if SNR is to be used as a performance metric for novel electrode designs. This standardization could include selecting or deriving general equations with standardized variables. Then, the SNR should be evaluated based on results from using specific electrode arrangement on a standardized synthetic skin that mimics the properties of the human scalp; as a synthetic skin can be replicated in distinct labs, this would allow research groups to have the same recording methods and variables when calculating SNR. Therefore, the synthetic skin could provide a standardized SNR metric to compare novel electrode performance across research groups, while the human subject could provide a real-world example of the electrode performance.

Finally, due to the previously discussed challenges, it is difficult to compare the performance of novel electrode designs for EEG recording. There have been publications focusing on designing an experimental paradigm to benchmark and compare various EEG acquisition systems [132], but these methods still have variability and do not focus on evaluating the electrode design itself. Thus, dimensional analysis methods could be used to discover a dimensionless parameter based on the electrode’s material properties that could indicate its effectiveness for noninvasive EEG recording. Work has been performed that can inform this analysis, such as the evaluation of the complete electrode model (CEM) for EEG simulation [133], which is a model originating from electrical impedance tomography (EIT) that factors in the electrode’s size, shape, and effective contact impedance. CEM has also been used with EIT methods to create a fluid flow visualization system, where fluid flows through a cylindrical domain, and current is injected through electrodes placed on the cylinder’s surface to obtain information about what is occurring in the flow [134]. A dimensional analysis was performed, factoring in the electrodes and characteristics of the domain, revealing relationships between the electrode properties and the domain. While there is not a one-to-one correlation between the previously mentioned work and the standardization of EEG electrode evaluation, it is highly probable that similar research could be performed to reveal a beneficial dimensionless parameter. This parameter could characterize the effectiveness of an EEG electrode and may reveal information about the nature of EEG recording on the scalp. Some of the key parameters for the dimensional analysis could include the electrode’s contact surface area, skin-surface contact impedance, and electrical conductivity. If the electrode is deformable and has a piezoresistive effect, then parameters should be included to factor in the deformability of the electrode, such as parameters extracted from hyperelastic material [135,136,137,138]. Parameters that characterize the domain that the electrode is recording on may need to be included as well. While this analysis is beyond the scope of this review report, it will be part of our future work.

## 6. Conclusions

The application of wearable biosensors, such as in electroencephalography (EEG) signal detection, has garnered significant interest. Carbon nanoparticles, integrated into biocompatible polymer matrices, have facilitated the development of nanocomposites with precisely tunable mechanical and electrical properties. These advanced materials have proven highly effective as wearable sensors for physiological signal acquisition, particularly in EEG, offering superior mechanical flexibility, electrical performance, and signal fidelity compared to commercially available alternatives while maintaining scalability and feasibility in production processes.

This review sought to deliver an exhaustive analysis of the recent advancements in the field, focusing on the design, fabrication techniques, and performance characteristics of carbon nanocomposite-based EEG electrodes. It aimed at identifying prevailing research trends and establishing a benchmark for the current state-of-the-art in carbon nanocomposite technology for high-resolution EEG monitoring. By synthesizing the existing literature, the review intended to guide the methodological approaches in future research, fostering the development of next-generation nanocomposites with optimized properties tailored for EEG applications. Furthermore, the review highlights current practices in evaluating novel EEG electrodes, emphasizing the critical need for standardized assessment protocols to enhance comparability and reliability in the field.

## Figures and Tables

**Table 1:** Summary of the general characteristics of carbon nanomaterials utilized across reviewed literature.

Carbon Nanomaterial	Dimensionality and Structure	Mechanical Properties	Electrical Properties
Single-walled Carbon Nanotubes	1D Tube	High Stiffness, Axial Strength [52]	Highly Conductive [52]
Multi-walled Carbon Nanotubes	1D Concentric Tubes	High Stiffness, Axial Strength [52]	Highly Conductive [52]
Carbon Nanofibers	1D Cylindrical Layers	High Mechanical Strength, Flexibility [53]	Highly Conductive [53]
Graphene	2D Hexagonal Lattice	High Stiffness [54]	Highly Conductive [55]
Carbon Black	3D Spherical Agglomerate	High Mechanical Strength [56]	Variable Conductivity [56]
Carbon Fibers	3D Cylinder	Axial Strength [57]	Conductive [57]

**Table 2:** Summary of CNT type, dimensions, and utilization in EEG electrode fabrication.

CNT Type	Dimensions (L × D) (μm × nm)	Utilization	Dispersion Method	Reference
SWCNT	- × 1–2	Surface layer	Spray-coating	[68]
MWCNT	-	Conductive filler	Mechanical mixing, sonication	[33]
MWCNT	-	Conductive filler	Sonication with solvent	[37]
MWCNT	-	Conductive filler	Mechanical mixing	[38]
MWCNT	100–200 × 6	Conductive filler	Paste mixer, milling	[39]
MWCNT	10–30 × 10–20	Conductive filler	Sonication with solvent	[15]
MWCNT	5–20 × 16 ± 3.6	Conductive filler	Pour-over	[41]
MWCNT	3–12 × 12	Conductive filler	Sonication with solvent	[60]
MWCNT	10–20 × 10–20	Conductive filler	Sonication with solvent	[62]
MWCNT	1–25 × -	Conductive filler	Milling, mechanical mixing	[65]
ACNT	400 × -	Surface layer	Dry spinning	[66]
CNT	-	Conductive filler	Mechanical mixing	[18]
CNT	-	Conductive filler	-	[51]
CNT	-	Conductive filler	Sonication with solvent	[61]
CNT	-	Conductive filler	Mechanical mixing	[63]
CNT	-	Conductive filler	Sonication with solvent	[64]
CNT	5–30 × 1–2	Surface layer	Spin-coating	[67]

**Table 3:** Summary of graphene synthesis, forms, and utilization in EEG electrode design.

Graphene Form	Synthesis	Utilization	Reference
Monolayer	CVD	Thin film	[13,79]
Bilayer	CVD	Thin film	[36,76]
Few-layer	CVD	Conductive layer	[32]
3D	Laser-induced	Thin film	[35,40,77]
3D	CVD	Conductive layer	[16]
Nanoribbons	Oxidation reduction	Conductive filler	[78]
Reduced GO	Chemical reduction	Conductive layer	[44,80]
Reduced GO	Thermal reduction	Conductive layer	[84]
GO	Hummer process	Conductive filler	[83]
Epitaxial Mono/Bilayer	PVD	Conductive layer	[81]
Epitaxial Mono/Bilayer	Catalytic alloy	Conductive layer	[82]
Fluid or Powder	Commercially available	Conductive filler	[14,34,42,43,67,85]

**Table 4:** Summary of the distribution of substrate materials selected for electrode fabrication and typical filler loading for each substrate across the reviewed research publications.

Substrate Material	Uses	Distribution	Filler Loading	Reference
PDMS	Composite matrix, thin film, spin-coating	31%	0.5–10 wt%	[15,17,32,33,37,38,39,41,60,62,64,65,66,67,80,88]
Si/Ti/Ta wafer	CVD, lithography, catalytic alloy	10%	-	[14,16,32,81,82]
SEBS	Composite matrix, Thin film, Spin-coating	8%	5 wt%	[18,36,40,79]
PVA	Composite matrix, Thin film	8%	0.5–1 ratio%	[66,78,83,85]
Silicone	Composite matrix	8%	1–11.8 wt%	[19,34,49,50]
Modified PDMS	Composite matrix	8%	1–4 wt%	[61,63,64,73]
PU	Thin film, insulator	8%	-	[35,38,68,77]
Nylon	Bristles, e-textiles, membranes	6%	-	[44,46,84]
PEDOT:PSS	Thin film	4%	-	[13,76]
Copper	Flexible laminate	4%	0–100%	[42,67]
Unspecified TPE	Composite matrix	2%	-	[87]
Cotton	E-textiles	2%	-	[43]
EPDM	Composite matrix	2%	45 wt.%	[51]

**Table 6:** Summary of the reported signal-to-noise ratios for both carbon-based nanocomposite electrodes and commercial wet Ag/AgCl electrodes.

Electrode Type	Measured SNR, Carbon-Based (dB)	Measured SNR, Commercial Ag/AgCl (dB)	SNR Equation	Reference
Ag/CNT/PDMS Composite	26.83	25.23	-	[41]
GPG Thin-film	23.9	21.8	Equation (1)	[76]
SLPP Thin-film	35.78	19.8	Equation (1)	[40]
CMSA Composite	13.74	14.03	-	[15]
VDE Composite	∼34	∼33	-	[73]
CNT/SEBS Composite	3.41	3.09	-	[18]
ACNT Thin-film	21.22 ± 0.74	21.57 ± 0.57	-	[66]
Ag/CNT-GO-PDMS Composite	∼90	∼80	Equation (2)	[67]
FLG/TiO_2_ Wafer	76.8	-	Equation (2)	[32]
EG/SiC Wafer	5–25 ± 5	30 ± 5	Equation (5)	[81]
BVNG Electrode	6–8	-	Equation (2)	[16]
GO/GL/PVA Thin-film	7.4	3.3	-	[83]
LSG/PU Thin-film	14.1	10.7	-	[77]
Cu-TiO_2_-CNT@PDMS	9.6–11.6	8.9–12.8	Equation (2)	[33]
PTG Thin-film	23	19	-	[13]
EARtrodes	∼21	∼21	-	[49]
Graphene/GO	20–30	20–55	Equation (4)	[34]
GFG Composite	30	-	-	[79]
AgNW-GES Thin-film	16.7	16	-	[35]
rGO Thin-film	3.9–16.8	-	-	[80]
Mo-BLG Thin-film	35.4	37.2	-	[36]
Silicone Composite	1–2	-	Equation (3)	[19]
CNT/aPDMS Composite	3.71	-	-	[63]

## Data Availability

Other supporting information or data could be made available upon reasonable request from the corresponding author.
